# Age-Related Differences in Susceptibility to Carcinogenesis: A Quantitative Analysis of Empirical Animal Bioassay Data

**DOI:** 10.1289/ehp.6871

**Published:** 2004-05-12

**Authors:** Dale Hattis, Robert Goble, Abel Russ, Margaret Chu, Jen Ericson

**Affiliations:** ^1^George Perkins Marsh Institute, Clark University, Worcester, Massachusetts, USA; ^2^Office of Research and Development, U.S. Environmental Protection Agency, Washington, DC, USA

**Keywords:** carcinogenesis, fetal, ionizing radiation, mutagenic chemicals, risk assessment, statistical analysis, susceptibility

## Abstract

In revising cancer risk assessment guidelines, the U.S. Environmental Protection Agency (EPA) analyzed animal cancer bioassay data over different periods of life. In this article, we report an improved analysis of these data (supplemented with some chemical carcinogenesis observations not included in the U.S. EPA’s original analysis) and animal bioassay studies of ionizing radiation. We use likelihood methods to avoid excluding cases where no tumors were observed in specific groups. We express dosage for animals of different weights on a metabolically consistent basis (concentration in air or food, or per unit body weight to the three-quarters power). Finally, we use a system of dummy variables to represent exposures during fetal, preweaning, and weaning–60-day postnatal periods, yielding separate estimates of relative sensitivity per day of dosing in these intervals. Central estimate results indicate a 5- to 60-fold increased carcinogenic sensitivity in the birth–weaning period per dose ÷ (body weight^0.75^-day) for mutagenic carcinogens and a somewhat smaller increase—centered about 5-fold—for radiation carcinogenesis per gray. Effects were greater in males than in females. We found a similar increased sensitivity in the fetal period for direct-acting nitrosoureas, but no such increased fetal sensitivity was detected for carcinogens requiring metabolic activation. For the birth–weaning period, we found an increased sensitivity for direct administration to the pups similar to that found for indirect exposure via lactation. Radiation experiments indicated that carcinogenic sensitivity is not constant through the “adult” period, but the dosage delivered in 12- to 21-month-old animals appears a few-fold less effective than the comparable dosage delivered in young adults (90–105 days of age).

Standard animal cancer bioassays were designed as a qualitative screen for carcinogenic activity. In this context, it is easy to see how the additional difficulties of dosing at early life stages might have been considered to provide an only modest incremental return of qualitative hazard identification information compared with the extra effort and complexity of assuring adequate and comparable delivery of test substances over a full lifetime of exposure, from conception through adulthood. Therefore, conventional animal cancer bioassay studies conducted by the U.S. National Toxicology Program (NTP) and elsewhere have been designed to start dosing in early adulthood—usually 6–8 weeks of age in mice and rats (NTP 1993, 1999).

Over the last couple of decades, however, animal bioassay results have been routinely used as a basis for quantitative projections of potential cancer risks for populations exposed over a full lifetime, from conception through death. Moreover, the results of such risk projections are routinely used to arrive at a variety of types of determinations needed for practical decisions, for example:

- How extensive is the cleanup that is needed at hazardous waste sites to achieve risks that are below *X* incidence of harm with *Z* confidence? [Hattis and Anderson 1999; U.S. Environmental Protection Agency (EPA) 2001]
- What health prevention benefits should be expected from reducing exposures by various amounts for toxicants in ambient air, drinking water, and foods subjected to the chemical transformations from different methods of cooking? Do the incremental benefits of specific intervention measures justify their costs, when compared with available alternatives? [National Research Council 2002; Office of Management and Budget (OMB) 2000].

In the current revision of cancer risk assessment guidelines by the U.S. EPA (2003a), a question has arisen about whether human exposures during early life stages—during adolescence and before—should be attached any greater weight in risk projections than exposures during adulthood that are analogous to exposures represented in conventional animal bioassay testing. After reviewing an extensive set of nonconventional animal bioassay testing results, the U.S. EPA (2003b) concluded that there was appreciable evidence that juvenile exposures to mutagenic carcinogens conferred greater risks per day of dosing than do exposures during adulthood. The U.S. EPA proposed that for mutagenic chemicals, exposures in the first 2 years of life should be assumed to be 10 times as potent as exposures in adulthood. A similar 3-fold increase in expected risk was proposed for assessments of the effects of exposures between 2 and 15 years of age.

Both the age cutoffs used in this proposal and the extent of the assumed increase in sensitivity relative to adults were the products of relatively informal analyses of the assembled database. There was no analysis of data for carcinogenesis after transplacental exposure in the fetal period, and there was no distinction between preadult exposures before versus after weaning. Moreover, comparisons were done based on juvenile:adult ratios of raw cancer incidence (the fraction of animals observed to develop tumors) for comparably dosed animals. This potentially introduced distortions of two types: first, there was no allowance for tumor multiplicity (more than one effective tumor generation event per animal) in animal groups where a large fraction of the animals developed tumors, and second, the ratio analysis necessarily excluded data sets in which no tumors were observed in adult animals. In this article, we somewhat expand the database assembled by the U.S. EPA (2003b), and we present a more formal statistically weighted analysis of relative cancer potency in terms of cancer transformations per animal per unit dose for animals in different age groups, scaled to the highest experimental dose used either in adult animals or (if no fully adult animals were tested) the oldest age group of animals included in the experiment. We also derived separate summary relative potency estimates for the fetal, birth–weaning (approximately 21 days in rodents), and weaning–60-day periods. Where dosage spans multiple age groups, we used dummy variables to represent the observed tumor risk as the sum of cancer contributions from dosing in different periods. The data were analyzed in a series of subsets (mutagenic vs. nonmutagenic chemicals vs. radiation; male vs. female; liver vs. nonliver) to show how the results depend on various factors.

## Description of the Databases

An overview of the data is presented in Table 1. Experimental results described in detail by the U.S. EPA (2003b) were corrected in a few cases and supplemented as follows:

- We added esophageal tumors for diethylnitrosamine (DEN; Peto et al. 1984); liver but not esophageal tumors from this article were included in the U.S. EPA analysis (U.S. EPA 2003b). Additionally, we added control observations reported by Peto et al. (1991).
- The exposure time was corrected for some vinyl chloride groups; we also included additional control and comparison group information for 52-week exposures described by Maltoni et al. (1984).
- We consolidated 6,000 and 10,000 ppm exposure groups for vinyl chloride; both of these are far greater than saturating levels for the metabolic activation of this chemical. Results for control (zero-dose) groups were also consolidated in several cases.
- We added the results of a major single-dose study of *N*-nitrosomethylurea by Terracini et al. (1976) and data from several reports on carcinogenesis from ionizing radiation in rats and mice (Cahill et al. 1975; Castanera et al. 1971; Di Majo et al. 1990; Knowles 1985; Sasaki 1991).
- We deleted groups that did not show defined observations for controls (numbers of animals tested and numbers with tumors).

Data for two nonmutagenic chemicals (DDT and dieldrin) were eliminated from the analysis because of the complexity of the dosing protocol used. In these experiments, some groups were given gavage exposures, some direct dietary exposures, and some both in sequence. This rendered unambiguous calculations of comparable dosages for the different groups difficult.

The principal analyses maintain the subdivisions between continuous-dosing protocols (in which dosing was maintained at a given rate for a defined period) versus discrete-dosing experiments (in which only a single dose, or up to four single doses were given to the animals at defined ages).

The full databases as well as models used for the statistical analyses of continuous, discrete, and radiation dosing data are available on our website (Hattis 2004).

## Modeling Methods

### Dosimetric conversions.

The assessment of comparable dosimetry for animals in different life stages has been a substantial issue in discussions of the analysis of these data. For various experiments in the original U.S. EPA listing (U.S. EPA 2003b), doses are quoted in terms of a concentration in an environmental medium (parts per million in diet or water or air to the individual for exposures after weaning, and to the mother in the case of fetal and birth–weaning exposures); in other cases, doses that were directly administered to animals via intraperitoneal or other injections were originally expressed in terms of micrograms per kilogram body weight or similar units. For entry into our analysis, we left the doses expressed in terms of environmental media concentrations unchanged, but we transformed the doses expressed as micrograms per kilogram body weight into micrograms/(kilogram body weight)^0.75^ by multiplying by estimated individual body weights to the one-quarter power. [Body weights for this purpose were taken from Nomura (1976) for mice and from the NTP (1999) and Zhang et al. (2001) for rats.] The aim of this transformation was to use a dose metric that (to the extent possible with available information short of physiologically based toxicokinetic modeling) is expected to be approximately proportional to internal daily average systemic concentrations of the parent compounds or putative active metabolites for continuous dosing, or area under the concentration–time curve (AUC) for discrete dosing.

The basis for this approach is similar to the principal current basis for dosimetric conversions for interspecies projections of cancer risks: that risks are assumed to be similar across species if the internal time-integrated concentrations of active metabolites are similar across species. Similarity of internal time-integrated concentrations is assessed with the aid of observations that both bulk uptake and elimination processes tend to scale across species with metabolic rates—approximately in proportion to body weight to the three-quarters power (Boxenbaum 1982; Federal Council for Science, Engineering and Technology 1992; Travis and White 1988; Travis et al. 1990). We have recently found that a similar transformation reconciles clearance rates of drugs across age groups in humans—at least after a period of severely deficient clearance in the first few months of infancy. Table 2, documenting this result, is based on a new regression analysis of human data for pharmaceuticals and methods that have been previously described (Ginsberg et al. 2002; Hattis et al. 2003). We have not located a comparable set of *in vivo* clearance observations in rats or mice. The literature does contain several reports that indicate depressed liver-metabolizing activity in the neonatal period based on *in vitro* measurements of the activity of some liver enzymes (Basu et al. 1971; Macleod et al. 1972) and differences between the sexes in the maturation of metabolizing capabilities (with generally greater activity observed in males). To assess the possible influence of a neonatal deficit of either activating or detoxifying activity on our findings, in the “Results” we include comparative analyses of the single-dose data for apparent relative sensitivity at narrowly defined time windows—contrasting day 1 after birth with later periods before and after weaning. We performed these comparisons for the two carcinogens that are thought to be direct acting (not requiring metabolic activation) and for those that putatively need metabolic activation before directly DNA-reactive substances are generated. We also assessed differences in apparent life-stage–related sensitivity between the sexes.

For ionizing radiation exposures, we have chosen to leave the doses in units of absorbed energy—rads or grays. If the oxidative products generated by radiation are the actual carcinogenic agents, and if these are predominantly destroyed by metabolism-dependent processes that operate at rates that scale with metabolic rates, it is possible that achieving comparable integrated dose × time levels of the active agents might require the same (body weight)^0.75^ conversions as used for chemicals. Making such a transformation would tend to decrease the time-integrated dosage for the younger post-natal animals and therefore would tend to increase the assessed sensitivity per dose relative to adult exposures. As it happens, such a transformation would have brought the radiation results more closely into alignment with the results for mutagenic chemicals.

### Equation fit and statistical optimization.

One basic difference between our methodology and that used for these data by the U.S. EPA (2003b) is a transformation of the raw observations of tumor incidence in different groups into the estimated number of tumor transformations per animal. This corrects for the fact that researchers cannot usually distinguish between cases where one or more than one tumor was induced in a particular organ within a specific animal (or where more than one tumor would have been induced at the site studied had the animal lived to the end of the observation period). To accomplish this, we use the same Poisson transformation that has been traditionally used for the multistage and related statistical models of carcinogenesis.

The Poisson distribution is appropriate for processes that occur as the result of independent events where the number of possible events occurring in a particular unit of observation is unlimited. Our use of the Poisson distribution in this case derives from the basic fact that tumors start in individual cells (Fialkow 1997; Knudson, 1973, 1977). Each tumor is conceived to be an independent event arising as the result of the completion of the last stage mutation in one stem cell out of many other susceptible stem cells in a particular organ. It should be noted that this last-stage event will not generally have occurred during the preadult life stages that are the focus of our analysis, but the effects of these early life exposures will manifest as incremental tumors that occur during the life-long period of observation of the animals.

Fraction of animals with tumors


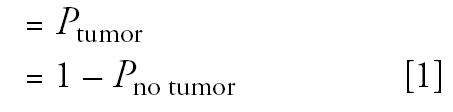



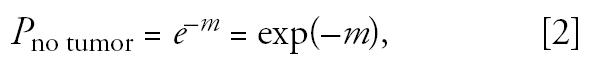


where *m* is the tumor transformations per animal at the studied site. Solving for *m*:


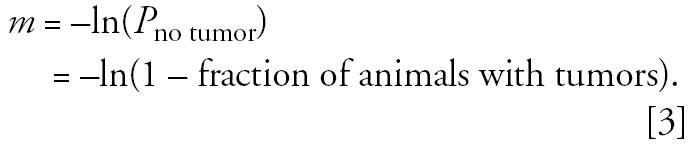


Because most of the experiments use only a single dose of carcinogen for each age group, no more sophisticated multistage treatment of tumor dose response is possible with these data. Given this, relative cancer transformation rates in different age groups in comparison with adult animals were estimated by fitting the continuous data to the following equation:

Fraction with tumors


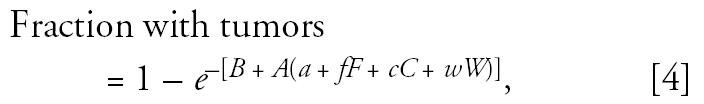


where *B* is the group background transformations per animal; *A* is the group adult transformations per animal at the highest adult dose rate; *a* is the fraction of the adult period with dosing at the maximum adult rate (this term reflects an adjustment where a group received less than the full adult dosing rate); *f* is the fraction of the fetal period with dosing at the maximum adult rate (also adjusted for dose rate as needed); *F* is the fetal:adult sensitivity ratio; *c* is the fraction of the birth–weaning period with dosing at the maximum adult rate (also adjusted for dose rate as needed); *C* is the birth–weaning:adult sensitivity ratio; *w* is the fraction of the weaning–60-day period with dosing at the maximum adult rate (also adjusted for dose rate as needed); and *W* is the weaning–60-day:adult sensitivity ratio.

In Equation 4, the terms designated with lowercase letters represent the input dosing and tumor response data for each group of dosed animals or controls. Where continuous daily dosing occurred over only part of a life stage, we entered the fraction of the life stage where dosing occurred. Similarly, where dosing for a particular group occurred at a fraction of the maximal rate given to adults, that fraction was entered as input data. This model form treats contributions to ultimate cancer transformation events from different life stages as additive.

The equation has two types of estimated parameters (designated with upper case letters). First, *A* and *B* are used only within specific experiments (a particular tumor type associated with exposure to a particular chemical in a particular animal group). By contrast, the three remaining “generic” parameters (*F*, *C*, and *W*) are estimated based on the results of all the dose groups for all chemicals and animals included in a particular run that contained some dosing within each life stage, compared with controls. Thus, for these generic parameters, the results represent summary central estimates [and upper (UCL) and lower confidence limits (LCL)] for all chemicals, tumor types, species (rats and mice), and other characteristics of the included experimental data. In light of this, in the “Results” we present alternative sets of estimates designed to explore the influence of sex, mutagenic character, tumor site, and other characteristics on the assessments of differences in susceptibility among life stages. Finally, because the doses used in the model fitting were expressed in terms of dose ÷ (body weight)^0.75^, the units of the relative sensitivity parameters should similarly be understood to be


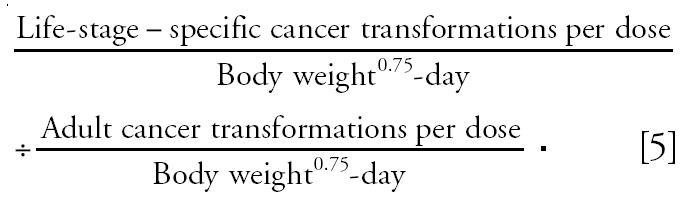


Estimates of the uppercase terms were derived by minimizing the “deviance” between observed and model predicted data points, as described by Haas (1994) and McCullagh and Nelder (1989): For nonzero numbers of tumors in a particular group the “deviance” is


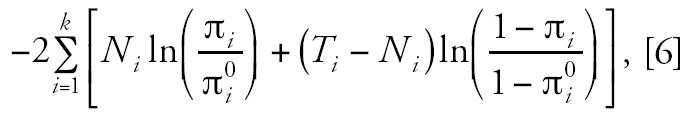


where *k* is the number of dose groups; *N_i_* is the number of animals with tumors in group *i*; *T_i_* is the total number of animals in group *i*; π*_i_* is the model-predicted proportion of animals with tumors in group *i*; and π^0^*_i_* is *N_i_*/*T_i_*.

This deviance-minimization optimization was accomplished in Microsoft Excel spreadsheets using the “solver” facility (Microsoft Corporation, Redmond, WA). Haas (1994) also provided procedures for deriving profile-likelihood-based confidence intervals (Venzon and Moolgavkar 1988) for these fitted parameters based on the chi-square statistic. For each confidence interval estimate, all parameters other than the one being assessed were allowed to vary. Thus, the upper and lower 95% confidence limits for the birth–weaning:adult sensitivity estimates reflect possible uncertainties in all the group background transformations per animal, group adult transformations per animal, and the sensitivities of fetal and weaning–60-day life stages relative to adults. A similar approach was used for the discrete dosing data and for the combined continuous and discrete data by dividing the doses by the estimated numbers of days in each dosing period (8 days for the fetal dosing period, 21 days for the birth–weaning life stage, 39 days for the weaning–60-day life stage, and 663 days for the adult period).

## Results and Discussion: Relative Sensitivity of Different Life Stages in Animals

Before considering the age-related differential sensitivity results for continuous versus discrete dosing in detail, it is worth noting that they may be reflecting somewhat different factors. The continuous dosing results:

- Include enzyme induction effects, if any
- Inherently reflect a dilution of any fluctuations in short-term sensitivity caused by, for example, waves of cell proliferation in specific organs in narrow time windows
- Possibly present fewer complications from high-dose kinetic and dynamic nonlinearities
- Have somewhat more straightforward implications for adaptation of traditional chronic dosing assessments.

On the other hand, the results from experiments where dosing was administered at discrete times:

- Almost always exclude direct enzyme induction effects
- Are capable of revealing short-term sensitivity fluctuations, to the extent that these occur
- Are likely to be done at somewhat higher dose rates, with some increase in potential complications from high-dose nonlinearities
- Have more straightforward implications for assessment of risks from acute exposure events.

### Results for overall continuous chemical, discrete chemical, and radiation dosing data sets.

Table 3 shows the results of fitting the continuous and discrete dosing data as a whole, together with similar results for radiation exposures. In all three sets of data, the birth–weaning period is suggested to be the most sensitive per day of dosing, followed by the fetal period and the weaning–60-day period. Each independent data set yields a central estimate of the birth–weaning sensitivity that is about 5- to 10-fold greater than the sensitivity per day of dosing in adulthood, with doses expressed per body weight^0.75^.

### Mutagenic versus nonmutagenic chemicals.

In the case of the continuous dosing data, some of the chemicals were classified by the U.S. EPA (2003b) as mutagenic, and some not. (All of the chemicals with discrete dosing data, and ionizing radiation, are mutagenic.) Table 4 shows the continuous dosing results broken out for mutagens versus nonmutagens. In contrast with the mutagens, for nonmutagenic carcinogens none of the age groups manifest significantly greater sensitivity than is seen for adults (defined as 1 in these tables). It should also be noted that separating out the nonmutagens leaves the mutagenic compounds showing significantly more birth–weaning period sensitivity than is seen for either the discrete-dosing chemical data or the radiation observations.

### Male versus female animals.

Tables 5–7 show the contrast between results in male versus female animals for continuously dosed mutagens, mutagenic chemicals delivered in discrete doses, and radiation experiments, respectively. The differences appear most prominent for the continuous dosing data (Table 5), where males seem to have much larger increases in sensitivity relative to adults for the fetal and birth–weaning life stages, and by contrast, females show a large increase in sensitivity for the weaning–60-day period. Considerable reserve is in order in interpreting the latter result, however, in the light of the slender database available for the continuous dosing analysis (only 3 chemicals and 16 dose groups for each sex) and the fact that neither the larger set of discrete-dosing data (Table 6) nor the radiation-dosing data (Table 7, based on fetal and weaning–60-day stages only) exhibits a similar enhanced female relative sensitivity for the weaning–60-day period, compared with males.

One way of weighing the different observations from continuous versus discrete chemical dosing experiments is to combine the two sets of results into a single model for analysis. The results of such a combination for male and female life-stage relative sensitivity ratios are shown in Table 8. The combined data tend to reinforce the suggestion that there are male–female differences in age-related sensitivity patterns but fail to sustain the initial suggestion from the continuous dosing data of an increase in the sensitivity for females in the weaning–60-day period relative to adults. On the other hand, the combined data do indicate an increased sensitivity for this period in males. The combined data for the fetal and birth–weaning periods indicate much more prominent excess sensitivity relative to adults in males than in females.

### Distributional form for the statistical uncertainties in estimated life stage/adult sensitivities.

**Figure 1:**
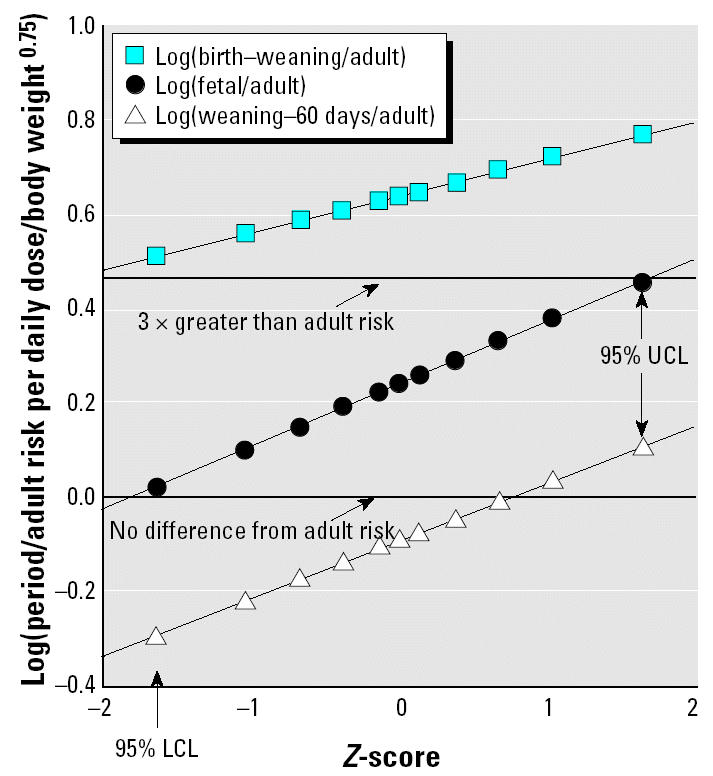
Lognormal plots of likelihood-based uncertainty distributions for cancer transformations per daily dose for various life stages for mutagenic chemicals (relative to comparable exposures of adults) for combined discrete and continuous dosing experiments in females. Log(birth–weaning/adult): *y* = 0.646 + 0.0785*x*; *R*^2^ = 1.000. Log(fetal/adult): *y* = 0.246 + 0.134*x*; *R*^2^ = 1.000. Log(weaning–60 days/adult): *y* = 0.0880 + 0.124*x*; *R*^2^ = 0.999.

**Figure 2:**
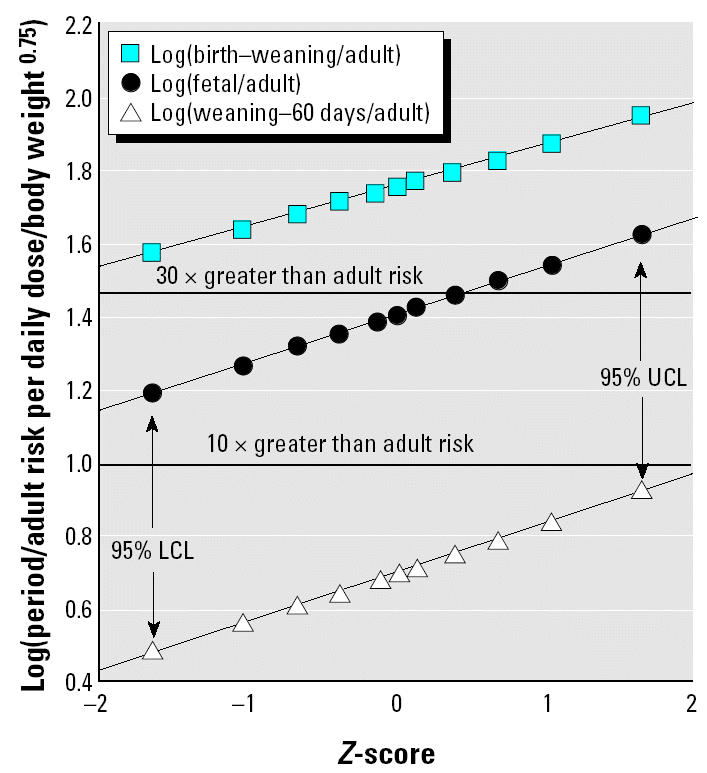
Lognormal plots of likelihood-based uncertainty distributions for cancer transformations per daily dose for various life stages for mutagenic chemicals (relative to comparable exposures of adults) for combined discrete and continuous dosing experiments in males. Log(birth–weaning/adult): *y* = 1.76 + 0.113*x*; *R*^2^ = 0.999. Log(fetal/adult): *y* = 1.41 + 0.132*x*; *R*^2^ = 1.000. Log(weaning–60 days/adult): *y* = 0.705 + 0.133*x*; *R*^2^ = 0.999.

Figures 1 and 2 show lognormal probability plots (Hattis and Burmaster 1994) of the statistical uncertainty distributions for the life stage:adult sensitivity ratios for the male and female combined discrete and continuous dosing data for mutagenic carcinogens. In this type of plot, correspondence of the points to the fitted line is an indicator of the fit of a log-normal distribution to the statistical uncertainties in central estimate life stage:adult sensitivity ratios. (The *Z*-score that makes up the *x*-axis is the number of standard errors above or below the median of the normal distribution log_10_ transformed values.) It can be seen that the uncertainty distributions are well described by the lognormal fits. We stress that these plots are of confidence limits on the aggregate central tendency results for all chemicals in the covered groups. The uncertainties in estimates for individual chemicals are being analyzed separately (Hattis et al., unpublished data), together with implications for human risk for a particular mutagenic chemical.

### Rats versus mice.

Table 9 shows comparative results for life-stage–specific relative tumor sensitivities in rats versus mice for the combined discrete and continuous dosing experiments. There is a suggestion that the rat data may indicate somewhat larger effects relative to adults for the fetal and weaning–60-day life stages; however, the 95% confidence limits overlap. In the light of the very limited numbers of chemicals with relevant observations for rats, there should be no strong inference that the suggested rat/mouse differences are real.

### Direct-acting carcinogens versus agents requiring metabolic activation.

All but two of the mutagenic carcinogens covered in the database are thought to require metabolic activation to produce DNA-reactive agents (U.S. EPA 2003). The two exceptions are the nitrosoureas—methyl- and ethylnitrosourea. Comparing life stage:adult sensitivity results for the metabolically activated versus direct-acting compounds can shed light on whether the previous results, including the relevant dosimetry, are likely to have been appreciably distorted by immaturity of metabolic activating systems in the neonatal period.

Table 10 shows the relevant comparison using our standard breakdown of life stages, based on the single-dose data. The results indicate a clear difference in fetal sensitivity for direct-acting versus metabolically activated compounds. As might have been expected, there is, if anything, less carcinogenic susceptibility in the fetal period for metabolically activated compounds, whereas the fetal life stage shows 5- to 25-fold greater sensitivity than adults for the direct-acting nitrosoureas.

Table 11 shows the results of using a finer breakdown of time periods, made possible by the focus on data resulting from direct dosing at discrete times. Beyond the fetal period, there is no apparent difference in the pattern of relative sensitivity with age between the nitrosoureas and the metabolically activated carcinogens. In both cases, relative sensitivity peaks near birth and declines progressively thereafter until it reaches about double the adult sensitivity at day 21. Beyond the fetal period, there is thus no indication of a perinatal deficit in metabolic activating activity for this set of carcinogens.

### Direct dosing in the birth–weaning period versus dosing via lactation.

Another important dosimetric issue is whether the lactational exposures resulting from primary dietary exposure to maternal animals are in fact equivalent to doses directly administered to pups during the birth–weaning period. Table 12 shows the results of separate estimations of the relative tumor susceptibility for direct versus lactational exposure for the combined set of continuous and discrete dosing experiments. The data show that no diminution in birth–weaning sensitivity is indicated for lactational exposures compared with direct administration of known doses. If anything, the lactational exposures appear somewhat more potent than direct administration per unit of estimated external exposure, although the 95% confidence limits overlap. One possible interpretation of this result, if repeated, is that some of the bolus doses given in the direct administration experiments may have partially saturated metabolic activation pathways, leading to less effective dose of DNA-reactive metabolites per unit exposure than when similar materials are administered more slowly via milk.

### Radiation results for different times during the “adult” period.

The “adult” comparison groups for the discrete chemical dosing experiments generally were exposed in early adulthood—within 4–6 months of age. By contrast, the radiation experiments include groups extending to much older ages—up to 16–18 months. As shown in Table 13, these data indicate a considerable reduction in sensitivity for radiogenic cancer induction with advancing age.

### Liver tumors versus tumors in other organs.

As indicated in Table 1, many of the tumors studied in these rodent experiments come from the liver, particularly for the continuous dosing studies. We have found that, in general, life-stage–specific enhancements of sensitivity seem to be greater for the liver than for the lung, but life-stage–specific excesses in sensitivity are still apparent for the aggregate of nonliver, nonlung organs (Hattis 2004).

### Toward quantitative applications in human health risk assessment.

On a qualitative level, this analysis provides more detailed understanding and confidence in the fact that there is an increased early-life sensitivity for mutagenic carcinogens—reinforcing the conclusions drawn by the U.S. EPA (2003b). The next step toward applying these data for quantitative human risk assessment is to develop time/age mapping between rodents and people. What ages in people approximately correspond to the rodent fetal, birth–weaning, and weaning–60-day periods studied in this analysis? We are developing a preliminary mapping based on the times at which rodents and people attain various fractions of the average body weights they have at sexual maturity (Hattis et al., unpublished data). In this second article we also use a Monte-Carlo model–based distributional analysis of the combined uncertainties in *a*) the central estimates of life-stage–related differences in carcinogenesis susceptibility, as derived in this article; *b*) the chemical-to-chemical variation in the life-stage–related susceptibility estimates; and *c*) the rodent/human time mapping uncertainty. Quantitative assessment of these three uncertainties together is needed for full distributional analyses of cancer risks for exposures in early life stages.

## Figures and Tables

**Table 1:** Overall description of the databases.

			Dose groups with exposures in specific life stages (no. of animals × tumor-site observations)				
Dosing protocol	No. of chemicals or radiation types	Total dose groups	Control groups	Fetal	Birth–weaning	Weaning–60 days	Adult (≥ 60 days)
Continuous	9 (5 mutagenic)*^a^*	151*^b^* (103 liver)	29 (2,562)	14 (820)	62 (3,071)	62 (6,128)	85 (7,544)
Discrete (1–4×)	6 (all mutagenic)*^c^*	274*^b^* (90 liver)	45 (2,926)	8 (290)	117*^d^* (4,681)	85*^d^* (3,596)	37 (979)
Radiation	4*^e^*	138 (42 liver)	21 (4,283)	18 (1,323)	18 (1,744)	18 (1,529)	63 (3,668)

In some experiments, tumor observations were reported separately for two or more anatomical sites (e.g., liver and stomach). In these cases, the numbers reported here count the same individual animals more than once.

a The chemicals classified as mutagenic were benzidine, benzo(*a*)pyrene, DEN, safrole, and vinyl chloride; the chemicals classified as not mutagenic were amitrole, diphenylhydantoin, ethylene thiourea, and polybrominated biphenyls.

b The numbers of groups do not add to the total because some groups had dosing in more than one life stage.

c Benzo(*a*)pyrene, DEN, dimethylbenzanthracene, ethylnitrosourea, methylnitrosourea, and urethane.

d Sixty-six groups were dosed on the first day after birth, 69 groups received exposures between days 1 and 21, 19 groups were dosed on day 21, and 68 groups were dosed between days 22 and 60; this finer breakdown is presented in the expanded-time analysis of the single-dose data in Table 11. The sum of these numbers exceeds the total because some groups received dosing in more than one of these more finely divided time categories.

e The ionizing radiation exposures were from ^137^Cs gamma rays, X rays, neutrons, and internal beta rays resulting from the injection of tritiated water.

**Table 2:** Geometric mean ratios*^a^* of child/adult clearance/body weight and (clearance/body weight^0.75^): regression results from 104 data groups for 27 drugs for humans in various age ranges.

Form for expressing total body clearance	Premature neonates	Full-term neonates	1 week–2 months	2–6 months	6 months–2 years	2–12 years	12–18 years
Mg/kg body weight	0.52*^a^* (0.43–0.63)	0.66 (0.61–0.73)	0.77 (0.71–0.84)	1.21 (1.06–1.39)	1.71 (1.52–1.92)	1.42 (1.31–1.53)	0.97 (0.78–1.2)
Mg/(kg body weight)^0.75^*^b^*	0.23 (0.19–0.28)	0.31 (0.28–0.34)	0.38 (0.35–0.42)	0.68 (0.59–0.78)	1.03 (0.91–1.17)	1.08 (1.00–1.17)	0.93 (0.74–1.17)

Data in parentheses indicate the ± 1 SE range.

a These data are the antilogs of the *B* coefficients that result from fitting the equation: log(mean clearance) = *B*0 (intercept) + *B*1 × (1 or 0 for chemical 1) + *B*2 × (1 or 0 for chemical 2) + … + *Ba* × (1 or 0 for age group 1) + *Bb ×* (1 or 0 for age group 2) + …. A more complete description of the underlying data and methodology has been reported by Ginsberg et al. (2002), Hattis et al. (2003), and Hattis (2004).

b Input clearance/(kg body weight)^0.75^ data for the regression results reported in this line were calculated from clearance/body weight data by multiplying by group mean estimated body weights0.25. For children ≥ 2 years of age, body weights for this transformation were estimated using the formulas described by Hattis et al. (2003), averaged for both sexes. Body weights of 2.5 and 3.5 kg were assumed for premature and full-term neonates < 1 week of age, respectively, and a log-linear interpolation was made between 3.5 kg at age 1 week and 6.3 kg at 2 months for groups with mean ages in that interval.

**Table 3:** Summary of results from fitting cancer bioassay data: relative susceptibility of different life stages per day of dosing.

Dosing type and age group	Maximum likelihood estimate	95% LCL	95% UCL
All continuous chemical dosing experiments*^a^*			
Fetal period (8 days beginning on GD12)	4.9	0.5	9.3
Birth–weaning (21 days)	8.7	6.5	10.8
Weaning–60-days (39 days)	0.000	0.000	0.24
All discrete chemical dosing experiments*^b^*			
Fetal period (8 days beginning GD12)	5.1	3.6	8.5
Birth–weaning (21 days)	10.5	7.2	16.2
Weaning–60-days (39 days)	1.51	1.03	2.31
All ionizing radiation dosing experiments*^c^*			
Fetal period (8 days beginning GD12)	3.5	2.2	5.7
Birth–weaning (21 days)	5.3	3.9	8.3
Weaning–60-days (39 days)	2.4	1.8	3.4

GD, gestation day. Data are maximum likelihood estimates and confidence limits of cancer inductions per dose/(body weight^0.75^-day) relative to comparably dosed adults.

a Based on a total of 151 group tumor incidence observations for nine chemicals.

b Based on a total of 274 group tumor incidence observations for six chemicals.

c Based on a total of 138 group tumor incidence observations for four radiation types.

**Table 4:** Comparative results for continuous dosing of chemicals classified as mutagenic versus those classified as nonmutagenic (U.S. EPA 2003b): relative susceptibility of different life stages per day of dosing.

Mutagenicity class and age group	Maximum likelihood estimate	95% LCL	95% UCL
Chemicals classified by the U.S. EPA as mutagenic*^a^*			
Fetal period	8.4	3.5	15.5
Birth–weaning	24	17.1	34
Weaning–60-days	3.7	0.0	9.1
Chemicals classified by the U.S. EPA as nonmutagenic*^b^*			
Fetal period	0.0	0.0	17.4
Birth–weaning	3.0	0.0	4.7
Weaning–60-days	0.0	0.0	2.0

Data are maximum likelihood estimates and confidence limits of cancer inductions per dose/(body weight^0.75^-day) relative to comparably dosed adults.

a Five compounds, 43 tumor incidence observations.

b Four compounds, 108 tumor incidence observations in animal groups.

**Table 5:** Comparative results for male versus female animals for mutagenic chemicals given in continuous dosing experiments.

Sex and age group	Maximum likelihood estimate	95% LCL	95% UCL
Male			
Fetal period	35	16.5	72
Birth–weaning	133	80	245
Weaning–60-days	0.0	0.0	9.7
Female			
Fetal period	2.3	0.24	9.7
Birth–weaning	3.4	1.1	8.4
Weaning–60-days	41	18	98

Data are maximum likelihood estimates and confidence limits of cancer inductions per dose/(body weight^0.75^-day) relative to comparably dosed adults, for continuous dosing for chemicals classified by the U.S. EPA (2003b) as mutagenic (three compounds, 16 tumor incidence observations).

**Table 6:** Comparative results for male versus female animals for mutagenic chemicals given in discrete dosing experiments.

Sex and age group	Maximum likelihood estimate	95% LCL	95% UCL
Male animals			
Fetal period	5.7	3.5	11.1
Birth–weaning	11.1	6.6	19.5
Weaning–60-days	1.58	0.99	2.6
Female animals			
Fetal period	4.4	2.1	10.2
Birth–weaning	9.7	5.6	20
Weaning–60-days	1.45	0.75	3.2

Data are maximum likelihood estimate and confidence limits of cancer inductions per dose/(body weight^0.75^-day) relative to comparably dosed adults, for discrete dosing for chemicals classified by the U.S. EPA (2003b) as mutagenic (six compounds, 137 tumor incidence observations).

**Table 7:** Comparative results for male versus female animals for radiation dosing experiments.

Sex and age group	Maximum likelihood estimate	95% LCL	95% UCL
Male animals*^a^*			
Fetal period	7.4	3.2	43
Birth–weaning	No data	No data	No data
Weaning–60-days	2.3	1.6	3.3
Female animals*^b^*			
Fetal period	2.7	1.5	5.4
Birth–weaning	4.7	3.4	8.7
Weaning–60-days	2.4	1.4	4.6

Data are maximum likelihood estimates and confidence limits of cancer inductions per dose in rads or grays relative to comparably dosed adults.

a Sixty-six tumor incidence observations for two forms of radiation (X rays and neutrons).

b Sixty-nine tumor incidence observations for three forms of radiation (gamma rays, neutrons, and internal exposure to beta rays from tritiated water).

**Table 8:** Comparative results for male versus female animals for mutagenic chemicals: analysis of combined data from continuous and discrete dosing experiments.

Sex and age group	Maximum likelihood estimate	95% LCL	95% UCL	Arithmetic mean
Male animals				
Fetal period	25	15.6	42	27
Birth–weaning	57	38	90	59
Weaning–60-days	5.0	3.1	8.6	5.3
Female animals				
Fetal period	1.77	1.05	2.9	1.83
Birth–weaning	4.4	3.3	6.0	4.5
Weaning–60-days	0.82	0.50	1.29	0.85

Data are maximum likelihood estimates and confidence limits of cancer inductions per dose/(body weight^0.75^-day) relative to comparably dosed adults (nine compounds, 153 tumor incidence observations).

**Table 9:** Comparative results for mice versus rats in combined discrete plus continuous dosing experiments.

Species and age group	Maximum likelihood estimate	95% LCL	95% UCL
Mice*^a^*			
Fetal period	6.5	4.2	9.9
Birth–weaning	17.7	13.2	24
Weaning–60-days	2.3	1.53	3.3
Rats*^b^*			
Fetal period	18.9	8.3	45
Birth–weaning	21	11.7	38
Weaning–60-days	3.9	1.94	7.3

Data are maximum likelihood estimates and confidence limits of cancer inductions per dose/(body weight^0.75^-day) relative to comparably dosed adults: discrete plus continuous dosing for chemicals classified by the U.S. EPA (2003b) as mutagenic.

a Eight compounds, 265 tumor incidence observations.

b Four compounds, 44 tumor incidence observations.

**Table 10:** Comparative results for discrete dosing of chemicals for direct-acting nitrosoureas versus other mutagenic carcinogens thought to require metabolic activation to DNA-reactive compounds: standard breakdown of life stages.

Metabolism class and age group	Maximum likelihood estimate	95% LCL	95% UCL
Direct-acting mutagenic carcinogens*^a^*			
Fetal period	11.6	5.4	25
Birth–weaning	10.2	5.1	21
Weaning–60-days	2.7	1.37	5.6
Metabolically activated mutagenic carcinogens*^b^*			
Fetal period	0.21	0.01	0.90
Birth–weaning	15.0	8.4	33
Weaning–60-days	1.24	0.76	2.3

Data are maximum likelihood estimates and confidence limits of cancer inductions per dose/(body weight^0.75^-day) relative to comparably dosed adults.

a Ethylnitrosourea and methylnitrosourea (108 tumor incidence observations).

b Benzo(*a*)pyrene, diethylnitrosamine, dimethylbenzanthracene, and urethane (166 tumor incidence observations in animal groups).

**Table 11:** Comparative results for discrete dosing of chemicals for direct-acting nitrosoureas versus other mutagenic carcinogens thought to require metabolic activation to DNA-reactive compounds: expanded breakdown of ages.

Metabolism class and age group	Maximum likelihood estimate	95% LCL	95% UCL
Direct acting mutagenic carcinogens*^a^*			
Fetal period	4.4	2.0	12.4
Day 1	6.2	3.6	18.0
Other birth–weaning (except 1 or 21 days)	3.7	1.8	10.0
Day 21	2.2	1.44	4.9
> 21 weaning–60-days	0.92	0.38	2.7
Metabolically activated mutagenic carcinogens*^b^*			
Fetal period	0.13	0.01	0.52
Day 1	17.3	10.0	36
Other birth–weaning (except 1 or 21 days)	10.7	6.2	22
Day 21	1.9	1.06	3.7
> 21 weaning–60-days	0.87	0.54	1.52

Data are maximum likelihood estimate and confidence limits of cancer inductions per dose/(body weight^0.75^-day) relative to comparably dosed adults.

a Ethylnitrosourea and methylnitrosourea (108 tumor incidence observations).

b Benzo(*a*)pyrene, diethylnitrosamine, dimethylbenzanthracene, and urethane (166 tumor incidence observations in animal groups).

**Table 12:** Effect of separate estimation of relative sensitivity in the birth–weaning period for lactational exposures versus direct administration: combined continuous and discrete dosing data for nine mutagenic carcinogens (317 tumor incidence observations).

Dosing mode and age group	Maximum likelihood estimate	95% LCL	95% UCL
Fetal period	6.0	5.5	8.8
Birth–weaning direct	11.6	8.5	16.1
Birth–weaning lactational	21.4	15.3	30
Weaning–60-days	1.70	0.77	2.4

Data are maximum likelihood estimates and confidence limits of cancer inductions per dose/(body weight^0.75^-day) relative to comparably dosed adults: discrete + continuous dosing for chemicals classified by the U.S. EPA (2003b) as mutagenic.

**Table 13:** Relative sensitivity for radiation-related carcinogenesis indicated by an expanded breakdown of adult age groups: all ionizing radiation dosing experiments (based on a total of 138 group tumor incidence observations for four radiation types).

Age group	Maximum likelihood estimate	95% LCL	95% UCL
Fetal period	2.1	1.3	3.4
Birth–weaning	3.1	2.2	4.8
Weaning–60-days	1.5	1.1	2.1
6–12 months	0.32	0.00	0.69
Elderly (19–21 months)	0.36	0.19	0.60

Data are maximum likelihood estimates and confidence limits of cancer inductions per rads or grays relative to young adults (90–105 days).
